# LASSI-L detects early cognitive changes in pre-motor manifest Huntington’s disease: a replication and validation study

**DOI:** 10.3389/fneur.2023.1191718

**Published:** 2023-07-18

**Authors:** Luis A. Sierra, Shelby B. Hughes, Clementina J. Ullman, Andrew Hall, Sarbesh R. Pandeya, Robin Schubert, Samuel A. Frank, Mark A. Halko, Jody Corey-Bloom, Simon Laganiere

**Affiliations:** ^1^Department of Neurology, Beth Israel Deaconess Medical Center, Boston, MA, United States; ^2^Department of Neurosciences, School of Medicine, University of California, San Diego, CA, United States; ^3^San Diego State University/UC San Diego Joint Doctoral Program in Clinical Psychology, San Diego, CA, United States; ^4^George-Huntington-Institute, Muenster, Germany; ^5^Harvard Medical School, Boston, MA, United States; ^6^Department of Psychiatry, McLean Hospital, Belmont, MA, United States

**Keywords:** semantic interference, cognitive, Huntington disease, executive function, pre-motor manifest HD, LASSI-L

## Abstract

**Background and objectives:**

Cognitive decline is an important early sign in pre-motor manifest Huntington’s disease (preHD) and is characterized by deficits across multiple domains including executive function, psychomotor processing speed, and memory retrieval. Prior work suggested that the Loewenstein-Acevedo Scale for Semantic Interference and Learning (LASSI-L)–a verbal learning task that simultaneously targets these domains - could capture early cognitive changes in preHD. The current study aimed to replicate, validate and further analyze the LASSI-L in preHD using larger datasets.

**Methods:**

LASSI-L was administered to 50 participants (25 preHD and 25 Healthy Controls) matched for age, education, and sex in a longitudinal study of disease progression and compared to performance on MMSE, Trail A & B, SCWT, SDMT, Semantic Fluency (Animals), and CVLT-II. Performance was then compared to a separate age-education matched-cohort of 25 preHD participants. Receiver operating curve (ROC) and practice effects (12 month interval) were investigated. Group comparisons were repeated using a preHD subgroup restricted to participants predicted to be far from diagnosis (Far subgroup), based on CAG-Age-Product scaled (CAPs) score. Construct validity was assessed through correlations with previously established measures of subcortical atrophy.

**Results:**

PreHD performance on all sections of the LASSI-L was significantly different from controls. The proactive semantic interference section (PSI) was sensitive (*p* = 0.0001, *d* = 1.548), similar across preHD datasets (*p* = 1.0), reliable on test–retest over 12 months (spearman rho = 0.88; *p* = <0.00001) and associated with an excellent area under ROC (AUROC) of 0.855. In the preHD Far subgroup comparison, PSI was the only cognitive assessment to survive FDR < 0.05 (*p* = 0.03). The number of intrusions on PSI was negatively correlated with caudate volume.

**Discussion:**

The LASSI-L is a sensitive, reliable, efficient tool for detecting cognitive decline in preHD. By using a unique verbal learning test paradigm that simultaneously targets executive function, processing speed and memory retrieval, the LASSI-L outperforms many other established tests and captures early signs of cognitive impairment. With further longitudinal validation, the LASSI-L could prove to be a useful biomarker for clinical research in preHD.

## Introduction

Huntington’s disease (HD) is an autosomal-dominant neurodegenerative disease that is characterized by a triad of progressive motor, cognitive, and behavioral abnormalities. The diagnosis of manifest HD is established by unequivocal motor signs, however, progressive cognitive impairment typically begins much earlier (1–4). Capturing cognitive deficits in the pre-motor manifest stage (preHD) remains critically important for detecting the earliest signs of decline and improving the precision of disease staging systems (5, 6).

The cognitive profile of HD has been extensively studied and is characterized by marked declines in executive functioning and processing speed (6), which affects multiple other domains (1–3, 6) and performance on many neuropsychological tests, including memory-based tasks (5, 7–10). For example, early manifest HD patients often exhibit more pronounced deficits during the retrieval rather than encoding phase on free-recall memory paradigms (11–15). In preHD, these deficits are more subtle and, depending on the cognitive assessment, may go undetected. For example, while the Wechsler Memory Scale failed to differentiate preHD from controls (16), other paradigms, such as verbal learning tests, were sensitive to these early changes (8, 17).

A pilot study by Sierra et al. (17) showed that the Loewenstein–Acevedo Scales for Semantic Interference and Learning (LASSI-L) (18)–a verbal learning paradigm specifically designed to elicit interference effects and intrusions during memory retrieval–captured robust differences between preHD and controls. Notably, the LASSI-L also appeared to outperform several commonly used tests in preHD–including Symbol Digit Modalities Test (SDMT) (19), Stroop Color Word Test (SWRT) (20), Trail Making Test A/B (TMT) (21), and semantic fluency (Animals) (17, 22). These preliminary results suggested that the LASSI-L could detect early cognitive changes in preHD. However, replication and validation studies had yet to be performed.

In this study, we sought to replicate and extend the findings previously reported by Sierra et al. (17) and to further analyze the sensitivity, validity and utility of the LASSI-L in preHD. Our approach was guided by the recommendations for the use and validation of cognitive scales in HD outlined by the International Parkinson and Movement Disorders Society (MDS) (10). Specifically, we administered the LASSI-L to larger preHD and control cohorts, which allowed for specificity, sensitivity and receiver operating curve (ROC) analyzes. We then compared preHD performance at Beth Israel Deaconess Medical Center (BIDMC) to an age and education-matched preHD cohort at University of California, San Diego, School of Medicine (UCSD) to determine whether the findings could be replicated across sites. We analyzed longitudinal changes in performance on the LASSI-L to investigate test–retest reliability and practice effects. We directly compared individual performance on LASSI-L to the California Verbal Learning Test (CVLT-II) (14), a similar paradigm previously used in HD. We then repeated these group comparisons using a smaller preHD cohort restricted to participants predicted to be early in the disease course (Far subgroup). Finally, to establish construct validity, we correlated LASSI-L performance with established neuroimaging biomarkers in preHD: volumetric decline in caudate, putamen, globus pallidus, and nucleus accumbens (23).

## Methods

### Participants

Participants with genetically-confirmed pathological expansion of mHTT (CAG ≥ 40) in the preHD stage and healthy controls (HC) were recruited at BIDMC in Boston, Massachusetts. The study protocol was reviewed and approved by BIDMC’s institutional review board. All individuals provided informed written consent before enrolling.

BIDMC inclusion criteria for preHD were: 18–65 years of age for all participants and confirmed genetic diagnosis of HD (i.e., ≥40 CAG repeats in the Huntingtin gene). HC participants were selected to match the preHD group across age, sex, and education to minimize confounding effects. Exclusion criteria for all cohorts included:

- UHDRS™ Total Motor Score (TMS) > 8, assessed clinically by a neurologist at all visits
- UHDRS™ Total Functional Capacity (TFC) <13
- UHDRS™ Independence score (IS) < 100
- other neurologic history, including stroke, seizure, and traumatic brain injury (defined as head trauma with loss of consciousness of >5 min or requiring treatment)
- medication regimens that were being actively changed or use of stimulant medication (eg, amphetamine salts/methylphenidate) or sedative (eg, opioid/benzodiazepine) <5 days before the study visit
- any current illicit substance use, remote alcoholism or frequent alcohol use (>14 drinks per week), bipolar disease, schizoaffective disorder, active suicidal ideation, history of psychosis, or concern for mild cognitive impairment/dementia

Additionally, participants at BIDMC underwent MRI safety screens and were excluded from the imaging portion of the protocol if they had any contraindication to MRI such as metal in the brain or implanted medical devices.

To validate our findings, we collaborated with the Huntington’s Disease Clinical Research Center at the University of California, San Diego (UCSD). This center had already incorporated the LASSI-L along with other neuropsychological assessments into their local data repository consisting of a distinct preHD cohort. The classification of preHD status at UCSD was derived from clinical data encompassing the Total Functional Capacity (TFC) scores and the Unified Huntington’s Disease Rating Scale™ (UHDRS™) Total Motor Score (TMS). To maintain consistency, we employed the same inclusion/exclusion criteria used for the BIDMC preHD cohort while selecting participants from the UCSD repository. UCSD data was anonymized with mean and standard deviation values shared electronically.

### Neurological and Q-motor assessment:

To ensure the preHD participants had not reached the manifest stage, a UHDRS™ motor exam was administered by trained neurologists at every visit (24). Participants who scored >8 on UHDRS™-TMS at any point were removed from the analysis. To further ensure that participants were not exhibiting subtle signs of motor disease, participants also underwent a speeded taping assessment using Quantitative Motor (Q-Motor) (25, 26) within 6 months of their baseline visit. Several measures on the Q-Motor, including speeded tap (e.g., mean/SD of inter-onset interval, non-dominant hand) have been shown to reliably detect early decline in motor control (25, 27–30). The Q-Motor speeded tap protocol has been described elsewhere (26). Briefly, participants were instructed to rapidly tap (a pressure transducer) with the index finger as quickly as possible for 10 s. After a practice round, speeded tap protocol was repeated 3 times with both the right and left index finger. Q-Motor raw sensor data were transferred to QuantiMedis at the George-Huntington-Institute in Münster, Germany and motor features were derived in blinded fashion by extracting tap characteristics (e.g., inter-onset-intervals, tap durations). Mean and standard deviation of features were computed per recorded trial and summarized as mean value over all trials for each recorded task, subject and visit (25, 26).

### Neuropsychological tests

We administered a battery of neuropsychological tests to both preHD and HC. These included the LASSI-L, Stroop Word Reading Test (SWRT, raw scores) (20), SDMT (raw scores) (19), Mini Mental State Exam (MMSE, raw scores) (31), TMT (raw scores) (21), Semantic fluency (Animals, raw scores) (22) and CVLT-II (raw scores) (32). The SWRT, Semantic Fluency, TMT and MMSE were selected because they are routinely used to study this population. SDMT was included because it is considered a landmark assessment in the new HD-ISS staging system (33). CVLT-II was selected to directly compare LASSI-L to an established verbal learning test.

### Loewenstein-Acevedo scale for semantic interference and learning

The LASSI-L is a unique verbal learning paradigm that uses semantic cueing as well as free recall and cued recall in alternating fashion to both maximize the number of initially encoded items and to elicit interference and intrusions in a time-restricted manner. The test uses two 15-word lists (A and B) with the same three semantic categories (fruits, articles of clothing, musical instruments). Administration of the LASSI-L is as follows: each item from List A is shown to the subject on a separate card and requires the subject to read the word aloud. After each presentation, the subject is prompted to recall all 15 words within 60-s using Free recall. The subject is then given each of the 3 semantic category cues - one at a time with 20-s per cue – and asked to recall all words from that category. The total number of correct items recalled after the first presentation for both free and cued recall is recorded under A1-Free recall and A1-Cued Recall. This process is repeated with the same list and scored under A2. The entire procedure is then repeated with an entirely different list of 15 words belonging to the same 3 categories (List B) to generate measures of B1 and B2. Once completed, the subject is prompted, without further presentation, to recall the original List A using both Free and Cued recall (termed A3). After a final 20-min delay, the subject is prompted to perform a Free recall of any item from either list (Delayed Recall). The LASSI-L probes for proactive semantic interference (PSI) by measuring the interference of learned List A on the initial ability to recall List B, therefore PSI = B1 cued. Failure to recover from PSI (frPSI) is captured by measuring recall ability after the second presentation of list B (frPSI = B2 cued). Similarly, retroactive semantic interference (RSI) is captured by measuring the interference of learned List B on the subsequent cued recall of List A (RSI = A3 cued). The final Delayed Free recall section (items from either A or B) captures the effects of combined interference throughout the test. The number of errors (intrusions) are recorded during all recall sections. Further information on methodology of the LASSI-L is available (17, 18).

### Longitudinal follow up

To track longitudinal changes in performance participants were assessed 3 times over 12 months (baseline, 6-month and 12-month visits). At baseline and at the 12-month visit, they underwent UHDRS™-TMS, SWRT, SDMT, verbal fluency, TMT (A/B), MMSE and LASSI-L. The same protocol was administered at the 6-month visit except CVLT-II was substituted for the LASSI-L to minimize confounding effects of administering two verbal learning tests at the same visit. Q-Motor was administered once within 6 months of the initial visit and again at 12 months.

### Imaging protocol

Scanning was performed on a 3 T GE SIGNA Premier XT MR scanner at BIDMC in Boston, MA using a Nova Medical 32-channel head coil. High-resolution three-dimensional T1-weighted structural scans were acquired for all participants with an Inversion Recovery Fast Spoiled Gradient Recall echo sequence (3D IR-FSPGR) using standardized protocols with the following parameters: TR = 7.252 ms, TE = 2.96 ms, TI = 400 ms, FA = 11°, FOV = 25.6 cm, matrix size of 256 × 256 × 256, and slice thickness of 1 × 1 × 1 mm, without slice gap. Given MRI safety exclusions (e.g., metal implants) and patient characteristics (e.g., claustrophobia), 47/50 participants (24 preHD and 23 HC) were able to complete the MRI protocol. Brain imaging was performed within 6 months of the baseline visit.

Freesurfer software (v.7.2) was used to automatically parcellate (Deskian/Killiany parcellation atlas) and segment subcortical volumes using the 3 T T1-weighted MRI described above (3D IR-FSPGR).^1^ The technical details of these procedures are described in prior publications (34). Volumetric analysis was restricted to brain regions that previously demonstrated early progressive atrophy in preHD: caudate, putamen, globus pallidus and nucleus accumbens (23). Left and right-sided brain volumes for each region (in mm^3^) were summed and individually normalized by dividing by estimated total intracranial volume. Group differences in volumes were compared using unpaired two-tailed t tests. Spearman rho was used to correlate brain volumes to performance on pre-selected test sections (i.e., PSI, PIE, delayed recall) and corrected with FDR < 0.05.

### Statistical analysis

Unpaired two-tailed *t* tests were used to analyze group differences in both demographics, Speeded Tap (Q-Motor), and neuropsychological test performance except for the MMSE and LASSI-L B1/B2 Intrusion sections, which were compared using Mann–Whitney U due to floor/ceiling effects and non-normal distribution. *χ*^2^ analysis was used to compare differences in categorical variables (sex). Effect sizes were measured using Cohen’s d. Type 1 errors were minimized on the CVLT-II analysis by multiple comparison testing using FDR < 0.05. For practice effects on LASSI-L, non-parametric analyzes were performed given smaller sample sizes: Spearman rho was used to correlate performance for all participants who completed both the first and last visit and Wilcoxon signed rank test was used to analyze group differences between both time points. Sample size for the final time point is smaller than baseline because of participant drop out (4 preHD, 4 HC) and because some participants have yet to reach the final visit (12 preHD and 13 HC).

To split the preHD cohort into “near to diagnosis” or “far from diagnosis” (Far subgroup), we used a CAG-Age Product Scaled (CAPs) score split of 0.85 (CAP = Age_0_ × (CAG − 33.66)/ 432.3326), which corresponded to 7.59 years from predicted onset (35). Unpaired t tests, Cohen’s d and FDR < 0.05 were used to compare the Far subgroup to HC on all cognitive tests.

To determine the ratio of intrusions to correct responses on the B1 and B2 cued recall sections, we used Percentage of Intrusion Errors (PIE) = total intrusion errors/(total intrusion errors + total correct responses). Correlations between Freesurfer volumes and cognitive testing scores on PSI, B1 Cued Recall Intrusions (total), PIE, delayed recall, SDMT, SWRT were assessed using Spearman rho, given the potential for both non-normality and non-linear relationship between atrophy and test performance. *p* values were corrected with FDR < 0.05. Given that LASSI-L performance declines with age and lower education (18), ANCOVA were performed using Group as the categorical variable, PSI as the dependent variable and Age/Education as covariates.

ROC using Youden’s J statistic and area under the ROC (AUROC) were calculated for PIE, PSI, SDMT and SWRT. Delong and bootstrap methods tests were used to compare PSI AUROC to the other three tests.

Individual caudate/putamen volumes, TMS and SDMT scores were entered into the new HD-ISS online calculator (33), which was derived from Tabrizi et al., to classify each preHD participant from Stage 0 to 4. In an exploratory analysis of PSI as a potential landmark assessment in the HD-ISS, we created a performance cutoff of 1.5 SD below the HC mean (as a Stage 2 sign of cognitive impairment) and reclassified each preHD participant. Statistical analyzes were performed in R 4.2.1 (R Foundation for Statistical Computing, Vienna, Austria).

## Results

Fifty participants (25 preHD and 25 HC) were enrolled in the study at BIDMC. Age range for this cohort was 25–65; mean age was 40.68 (SD = 11.12) for preHD and 36.84 (SD = 12.02) for HC. Mean education was 15.76 years (SD = 2.67) for preHD and 16.40 (SD = 2.31) for HC. 72% of the preHD group and 52% of the HC group were female. Mean CAG repeat length was 42.46 (SD = 2.28). Mean CAPs score was 0.79 (SD = 0.23). Mean TMS for the preHD group was 2.44 (SD = 2.68) and no participant had a TMS > 8 (Table 1). SWRT, MMSE, Semantic Fluency, and TMT-A group differences were statistically significant between preHD and HC (Table 1). In a subgroup analysis of the 45/50 participants who completed the Q-Motor speeded tapping (22 preHD and 23 HC), there were no group differences on tap duration variability (non-dominant hand) or mean inter-tap interval (non-dominant hand). Groups differences above (demographics, cognitive testing) remained unchanged when restricting the analysis to the 45 participants who completed Q-Motor.

**Table 1:** Demographics and baseline cognitive performance.

	Mean (SD)			*P* value (Cohen’d)	
	BIDMC preHD (*n* = 25)	UCSD preHD (*n* = 25)	BIDMC HC (*n* = 25)	BIDMC preHD vs. UCSD preHD	BIDMC preHD vs. HC
Age	40.68 (11.12)	42.28 (11.95)	36.84 (12.02)	0.626	0.247
Education	15.76 (2.67)	15.56 (1.92)	16.40 (2.31)	0.762	0.369
Sex (Female)	72%	56%	52%	0.239	0.145
CAG	42.46 (2.28)	42.16 (2.25)	---	0.676	---
TMS	2.44 (2.68)	2.00 (2.55)	---	0.555	---
CAPs, (mean)	0.79 (0.23)	0.80 (0.22)	---	0.876	---
SWRT	87.96 (16.08)	92.96 (23.81)	102.48 (13.09)	0.389	**0.001 (0.990)**
MMSE	28.36 (1.41)	28.00 (1.68)	29.32 (0.80)	0.395	**0.009 (0.837)**
SDMT	52.88 (10.49)	52.36 (14.70)	58.68 (13.79)	0.886	0.101 (0.473)
Semantic fluency (Animals)	22.39 (6.85)	---	26.40 (5.32)	---	**0.025 (0.654)**
TMT-A	31.48 (10.99)	---	23.04 (5.76)	---	**0.001 (0.962)**
TMT-B	58.20 (19.96)	---	54.56 (25.16)	---	0.574 (0.160)
Q-Motor TDSD^1^	0.29 (0.18)	---	0.24 (0.12)	---	0.372 (0.327)
Q-Motor IOMN^2^	0.14 (0.06)	---	0.14 (0.06)	---	0.973 (1.000)

CAG, (cytosine, adenine, and guanine); TMS, Total Motor Score; CAPs, CAG-Age Product Scaled; SWRT, Stroop Word Reading Test; MMSE, Mini-Mental State Examination; SDMT, Symbol Digit Modalities Test; TMT, Trail Making Test; Q-Motor, Quantitative Motor.

^1^Speeded-tapping tap duration variability, non-dominant hand.

^2^Speeded-tapping mean inter-tap interval, non-dominant hand.

Bold values are significant values to focus on.

**Figure 1:**
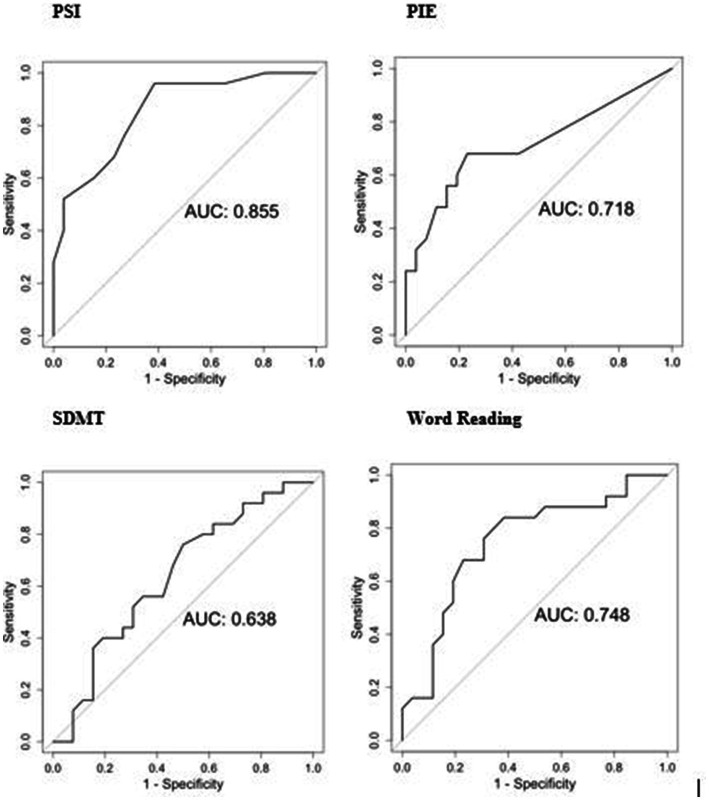
ROC analyzes of selected tests for BIDMC cohort (preHD vs. HC). PSI, Proactive Semantic Interference; PIE, percentage of semantic intrusion errors; SDMT, Symbol Digit Modalities Test.

BIDMC preHD and HC group differences on the LASSI-L can be seen on Table 2. Additionally, PIE was statistically significant for both PSI Intrusions (*p* = 0.004; *d =* 0.307), and frPSI intrusions (*p* = 0.025; *d =* 0.056). Using ANCOVA, PSI group differences remained significant after controlling for age [*F* (1,30) = 4.60, *p* = 0.046] and education [*F* (1,4) = 28.47, *p* < 0.0001]. AUROCs were: PSI = 0.855, PIE = 0.718, Stroop Word Reading = 0.748, and SDMT = 0.638 (Figure 1). AUROC comparisons were: PSI vs. PIE (*p* = 0.04); PSI vs. SDMT (*p* = 0.003); and PSI vs. Word Reading (*p* = 0.10). The correct words on the final Delayed recall section originated from both lists in balanced fashion (53% list A, 47% list B).

**Table 2:** LASSI-L performance comparison for BIDMC (preHD and HC) and UCSD preHD.

	Mean (SD)			P value (Cohen’s d)	
	BIDMC preHD (*n* = 25)	UCSD preHD (*n* = 25)	BIDMC HC (*n* = 25)	BIDMC preHD vs. UCSD preHD	BIDMC preHD vs. HC
A1 Cued Recall (correct responses)	10.36 (2.89)	9.40 (1.93)	12.00 (2.06)	0.203	**0.021 (0.654)**
A2 Cued Recall (correct responses)	13.40 (1.78)	13.16 (1.93)	14.36 (0.99)	0.650	**0.023 (0.667)**
B1 Cued Recall (PSI) (correct responses)	6.64 (2.87)	6.64 (2.18)	10.68 (2.32)	1.000	**0.0001 (1.548)**
B2 Cued Recall (*fr*PSI) (correct responses)	11.36 (2.72)	10.88 (2.52)	13.52 (1.81)	0.521	**0.002 (0.935)**
A3 Cued Recall (RSI) (correct responses)	9.32 (2.25)	8.40 (3.27)	11.32 (2.56)	0.252	**0.005 (0.830)**
Delayed Recall (correct responses)	19.20 (4.59)	18.88 (4.35)	24.24 (3.29)	0.801	**0.0001 (1.262)**
B1 Cued Recall Intrusions	1.64 (1.73)	2.48 (1.98)	0.52 (0.71)	0.119	**0.006 (0.847)**
B2 Cued Recall Intrusions	1.24 (1.36)	1.64 (1.85)	0.52 (0.82)	0.465	**0.026 (0.641)**
PIE Cued B1 (range:0.00–0.80)	0.20 (0.20)	0.05 (0.07)	0.26 (0.19)	0.219	**0.004 (0.307)**
PIE Cued B2 (range:0.00–1.00)	0.14 (0.21)	0.04 (0.05)	0.13 (0.14)	0.844	**0.025 (0.056)**

Range is specific to BIDMC.

PSI, Proactive Semantic Interference; frPSI, Failure to Recover from Proactive Semantic Interference; RSI, Retroactive Semantic Interference. PIE, percentage of semantic intrusion errors.

Bold values are significant values to focus on.

Performance on LASSI-L was compared to CVLT-II for all participants who completed both tests (*n* = 33, 16 preHD and 17 HC). Significant differences between preHD and HC were observed for the following sections of CVLT-II: List A Trial 1–5 (total), List A Short Delay, List A Short Delay Cued Recall Total, and Trial B Recall; however, only Trial B Recall survived FDR < 0.05 (Table 3). A subsequent comparison of LASSI-L to CVLT-II was repeated using only the preHD participants in the Far subgroup, using CAPs score cutoff <0.85 (> 7.59 years from diagnosis). PreHD Far subgroup (n = 13) characteristics were: age 37.7 years (SD = 11.6), education 15.23 years (SD =3.11), TMS 1.69 (SD = 2.06) and CAPs 0.66 (SD = 0.15) The only significant group differences between Far subgroup and HC for any cognitive test included in our battery were for LASSI-L PSI (*p* = 0.003; *d* = 1.174) and MMSE (*p* = 0.013; *d* = 0.950); however, only PSI survived FDR < 0.05 (Table 4).

**Table 3:** Performance on CVLT-II for preHD vs. HC.

	Mean (SD)			
	BIDMC preHD (*n* = 16)	BIDMC HC (*n* = 17)	*P* value (FDR-adjusted)	Cohen’s d
List A Trial 1–5; Total (correct responses)	52.06 (12.40)	61.24 (11.56)	0.035 (0.079)	0.766
List A Short Delay (correct responses)	10.56 (3.95)	13.41 (2.67)	0.021 (0.095)	0.845
List A Short Delay Cued Recall Total (correct responses)	11.56 (3.88)	14.12 (2.09)	0.024 (0.072)	0.821
List A Long Delay Cued Recall (correct responses)	12.00 (3.78)	13.82 (2.32)	0.136 (0.204)	0.584
List A Long Delay (correct responses)	10.63 (4.15)	13.18 (2.72)	0.063 (0.133)	0.727
List A Long Delay Recognition (total hits)	16.13 (8.90)	15.41 (0.87)	0.744 (0.744)	0.114
List A Delay Forced Choice Recognition (total accuracy)	15.75 (0.77)	15.94 (0.24)	0.340 (0.437)	0.333
Trial B Recall (correct responses)	5.44 (1.93)	8.12 (2.32)	**0.001 (0.009)**	**1.256**
Trial B (Total Intrusions)	0.25 (0.45)	0.12 (0.33)	0.340 (0.383)	0.329

Control cohorts are only from BIDMC.

Bold values are significant values to focus on.

**Table 4:** “Far from HD diagnosis” cohort demographics and cognitive performance.

	HD (*n* = 13)	HC (*n* = 17)	*P* value (FDR-adjusted)	Cohen’s d
Age	37.69 (11.59)	31.80 (12.49)	0.386	---
Education	15.23 (3.11)	16.13 (2.22)	0.311	---
TMS	1.69 (2.06)	---	---	---
CAPs	0.66 (0.15)	---	---	---
Caudate Volume (normalized)	0.428 (0.071)	0.516 (0.060)	**0.0003**	1.339
Putamen Volume (normalized)	0.619 (0.120)	0.745 (0.076)	**0.0005**	1.254
B1 Cued Recall (PSI) (correct responses)	6.77 (3.14)	10.29 (2.85)	**0.003 (0.03)**	**1.174**
B1 Cued Recall Intrusions	1.31 (1.49) 94% (16/17)*	0.71 (0.92) 100% (12/12)	0.184 (0.307)	0.485
PIE Cued B1	0.20 (0.23)	0.07 (0.09)	0.045 (0.150)	0.744
CVLT-II Trial B (correct responses)	6.08 (3.15)	8.12 (2.32)	0.050 (0.100)	0.737
TMT-A	28.54 (11.12)	24.29 (5.93)	0.189 (0.270)	0.477
TMT-B	57.46 (23.88)	54.18 (20.76)	0.690 (0.767)	0.147
SDMT	56.08 (10.63)	57.12 (15.03)	0.834 (0.834)	0.080
Word reading	88.62 (17.92)	102.53 (18.29)	0.047 (0.118)	0.768
Animals	23.08 (7.37)	25.65 (5.63)	0.287 (0.359)	0.392
MMSE	28.62 (1.04)	29.47 (0.72)	0.013 (0.065)	0.950

TMS, Total Motor Score; CAPs, CAG-Age Product Scaled; PSI, Proactive Semantic Interference; TMT, Trail Making Test; SDMT, Symbol Digit Modalities Test; MMSE, Mini-Mental State Examination, PIE, percentage of semantic intrusion errors.

Bold values are significant values to focus on.

The UCSD preHD comparison cohort had the following characteristics: mean age 42.28 years (SD = 11.95), mean education 15.56 years (SD = 1.92), 56% female, CAG repeat length 42.16 (SD = 2.25), CAPs 0.80 (SD = 0.22). The BIDMC and UCSD cohorts were matched with respect to age, education, sex, CAG repeats, CAPs and TMS (Table 1). There were no group differences on SWRT, MMSE, and SDMT (Table 1). There were no group differences between preHD cohorts on any section of the LASSI-L (Table 2).

As of 3/15/2023, 17 participants (9 preHD and 8 HC) have completed the final 12-month visit. For preHD subjects in this subgroup, there were no significant changes in TMS score (*p* = 0.766; Table 5). For the entire subgroup (preHD and HC), there were no group differences in LASSI-L performance between timepoints; scores on several sections of the LASSI-L were highly correlated: PSI (*r* = 0.88; *p* = < 0.00001), frPSI (*r* = 0.80; *p* = 0.0001), RSI (r = 0.69; *p* = 0.002), and Delayed Recall (*r* = 0.68; *p* = 0.002; Table 5). Similarly, Q-Motor measures were highly correlated between timepoints (Table 5).

**Table 5:** Longitudinal analysis and test–retest reliability for all participants at BIDMC (preHD and HC).

	Mean (SD)			
	BIDMC Baseline (*n* = 17)	BIDMC 12 month follow-up (*n* = 17)	Spearman rho, (*p*)	Wilcoxon signed rank, *p*
TMS	2.56 (3.24)	2.22 (3.35)	---	0.766
B1 Cued Recall (PSI) (correct responses)	8.24 (3.42)	8.12 (3.81)	**0.88 (< 0.00001)**	0.757
B2 Cued Recall (frPSI) (correct responses)	12.35 (2.37)	12.35 (2.69)	**0.80 (0.0001)**	0.904
A3 Cued Recall (RSI) (correct responses)	9.76 (2.73)	10.35 (3.26)	**0.69 (0.002)**	0.407
Delayed Recall (correct responses)	20.76 (4.69)	22.29 (5.67)	**0.68 (0.002)**	0.184
B1 Cued Recall Intrusions	1.06 (1.68)	0.65 (1.37)	0.24 (0.361)	0.705*
Q-Motor TDSD^1^	0.24 (0.19)	0.25 (0.19)	**0.684 (0.002)**	0.582
Q-Motor IOMN^2^	0.09 (0.02)	0.09 (0.01)	**0.950 (<0.00001)**	0.171

^*^The distribution of intrusions was insufficient for the Wilcoxon statistic, therefore a Signed Rank was used.

PSI, Proactive Semantic Interference; frPSI, Failure to Recover from Proactive Semantic Interference; RSI, Retroactive Semantic Interference.

^1^Speeded-tapping tap duration variability, non-dominant hand.

^2^Speeded-tapping mean inter-tap interval, non-dominant hand.

Bold values are significant values to focus on.

The theoretical performance threshold for PSI, (1.5 SD below HC mean = 10.65), was 7.20, indicating that 15/25 preHD (60%) and 3/25 (12%) controls were impaired on this test (Supplemental Figure S3).

### Imaging

Group differences between preHD and HC in four Freesurfer-derived brain volumes were significant in the following regions: caudate (*p* < 0.0001), putamen (p < 0.0001), nucleus accumbens (*p* = 0.0014) and globus pallidus (*p* = 0.028; Table 6). Intrusions on B1 Cued Recall (PSI section) were negatively correlated with both caudate volumes (spearman rho = − 0.51; two tailed *p* = 0.010) and putamen (spearman rho = − 0.40; two tailed *p* = 0.053). PIE was negatively correlated with caudate (spearman rho = − 0.40; two tailed *p* = 0.054) and putamen (spearman rho = − 0.40; two tailed *p* = 0.055). Correlation between Intrusions on B1 Cued Recall (PSI) and caudate survived FDR < 0.05 (*p* = 0.040). PSI and Delayed recall sections of LASSI-L, SDMT and SWRT were not significantly correlated with these regions (Supplementary Table S7).

**Table 6:** Brain volumes (normalized by estimated total intracranial volume*).

	HC (*n* = 23)	preHD (*n* = 24)	*P* value
Caudate	5.09 (0.61)	4.28 (0.64)	**<0.0001**
Putamen	7.35 (0.76)	6.14 (1.05)	**<0.0001**
Globus Pallidus	2.75 (0.23)	2.58 (0.23)	**0.028**
Accumbens	0.45 (0.13)	0.39 (0.14)	**0.001**

* Values = {[Right + Left brain volume (mm3)]/ total estimated total intracranial volume} x 1,000.

Bold values are significant values to focus on.

HD-ISS calculator (see footnote 2) revealed that the 24 BIDMC preHD participants would have been classified as: Stage 0 (CAG > 40 without biomarker of pathogenesis) = 11; Stage 1 (biomarker of pathogenesis without clinical signs or symptoms) = 8, and Stage 2 (biomarker of pathogenesis with clinical signs or symptoms) = 3. Two participants could not be classified, based on atypical discrepancies between brain volumes and motor/cognitive score. Using a cutoff of PSI < 7.20 (derived above, Supplementary Figure S3) as a sign of cognitive impairment, 4/8 participants in Stage 1 would have been reclassified as Stage 2. Additionally, 6/11 participants in Stage 0 would already be showing impairment on this test.

## Discussion

In this study, we showed that the LASSI-L captured early cognitive deficits in preHD and outperformed several established assessments in HD, thereby extending the primary findings reported in 2023 by Sierra et al. (17) These results were reliable across different sites and examiners and in a longitudinal analysis, the LASSI-L did not show significant practice effect after a 12-month interval. The PSI section demonstrated an excellent AUROC that was significantly better than SDMT, indicating that it could effectively discriminate preHD from healthy controls. Finally, a significant correlation between the number of intrusions on PSI and an established neuroimaging biomarker in preHD - caudate atrophy-further confirmed the construct validity of this assessment.

The current study confirms that two sections of the LASSI-L are remarkably sensitive to early changes in HD: PSI (*p* = 0.0001; *d* = 1.548) and delayed recall (*p* = 0.0001; *d* = 1.262) (17). PSI measures the inference of list A on the initial cued recall of list B. In multiple preHD cohorts, PSI has repeatedly exhibited large effect sizes indicating that a susceptibility to interference on memory tasks may be one of the earliest and most reliable deficits in this population. The sensitivity of the PSI is likely secondary to the simultaneous demands this section exerts on cognitive domains that are central to early dysfunction in preHD (6): executive function is challenged by list interference, the need to inhibit cued intrusions, and the need to retrieve information; processing speed is challenged by short timed sections; memory is challenged by the need to encode two supra-span lists.

The final delayed recall section exhibited the next largest effect size (Cohen’s d = 1.262). Sierra et al. (17) had previously interpreted this finding to be the result of combined proactive and retroactive interference (i.e., list A on list B and vice versa). In the preHD group, correct responses on the final delayed recall section originated from both lists in equal fashion (53% list A, 47% list B), lending support to this interpretation. In the current study, significant group differences were now also observed on many other sections: A1 Cued Recall, A2 Cued Recall, frPSI, RSI, and Intrusions for both PSI and frPSI. The larger replication study was likely adequately powered to uncover small but significant effects on these sections as well.

The LASSI-L detected changes in a preHD cohort that was at an early stage in the disease process, which was confirmed by a low mean UHDRS™-TMS score (2.2), a low mean CAPs score (0.79) and Q-Motor scores that were similar to healthy controls. In a subgroup analysis restricted to preHD subjects predicted to be far from diagnosis, the LASSI-L was, after controlling for multiple comparisons, the only cognitive assessment that detected significant differences with healthy controls (Table 4). The LASSI-L’s ability to detect early changes in this Far subgroup further highlights its potential utility as a marker of early change.

The Huntington’s Disease Integrated Staging System (HD-ISS) online calculator (see footnote 2) revealed that the majority of Stage 0 participants were already showing signs of significant cognitive impairment on PSI and that substituting LASSI-L for SDMT could also have shifted 50% of Stage 1 participants to Stage 2. These results further suggest that deficits on PSI occur early in the disease process. Incorporating the LASSI-L as a landmark assessment into future iterations of such staging systems could help delineate substages or provide an additional reliable endpoint for clinical trials targeting these early periods.

An important consideration when using cognitive tests as endpoints is the potential for practice effects upon repeated exposure. Tests associated with significant practice effects can obscure clinical deterioration, which can increase sample size requirements or the time required to observe the effect of an intervention (36). In a small subgroup analysis of participants who were able to complete the 12 month study, several sections of the LASSI-L, most notably PSI, appeared to demonstrate good initial test–retest reliability over 12 months (*r* = 0.88) without clear practice effects (Table 5). Importantly, these results mirrored the lack of significant clinical decline, as assessed by TMS and Q-Motor (Table 5). Although this finding is consistent with prior reports of LASSI-L reliability (37), larger samples will be required to firmly confirm this trend.

The LASSI-L shares certain features with verbal learning tests commonly used in HD. To investigate the relative sensitivity of each paradigm without creating confounds, we substituted the CVLT-II for the LASSI-L at the 6 month visit. In our study, sections of both tests demonstrated an ability to capture interference effects in the preHD group (LASSI-L: PSI, frPSI, RSI and CVLT-II: Trial B, Tables 2, 3), suggesting that verbal learning paradigms that elicit strong interference effects (proactive and retroactive) may be more likely to detect the earliest cognitive changes in preHD.

Finally, it is useful to note that the PSI and associated errors (B1 intrusions) are recorded within the first 8 min of the test, indicating that future iterations of the LASSI-L could be significantly shortened without compromising test integrity. This feature may be particularly valuable for clinical trials that seek to limit participant fatigue.

Existing literature extensively documents memory impairments associated with premanifest or early-stage Huntington’s disease (HD) (6, 38–42). These impairments primarily stem from executive function deficits that manifest in various abnormalities, including utilization of passive learning strategies (43), and difficulties with source memory (38). Other studies have also indicated that individuals with HD are more susceptible to interference effects on the temporal order of closely presented stimuli (44) and exhibit perseveration during paired-associate learning (45) and extra-dimensional shift learning (46). These deficits may provide insights into why the LASSI-L PSI section, which requires disregarding or inhibiting recently encoded items from an initial list within a brief time frame, emerges as the most sensitive section for this population.

Considerable evidence supports the effectiveness of verbal learning tasks in detecting early changes in individuals with HD. Earlier studies conducted before genetic testing for HD was available yielded mixed results in the preHD population. However, a notable study utilizing the PREDICT-HD dataset (47) demonstrated significant differences in HVLT-R performance between preHD individuals and controls as early as DCL1 (minimal motor abnormalities). While their study did not find differences in the DCL0 group (corresponding to the preHD Far group in our study), the authors suggested that this may be due to ceiling effects in the selected measures and recommended the use of more challenging memory assessment tools.

The combination of short timed sections, cueing (which optimizes initial encoding of list A but induces interference/intrusions on list B), and repeated list switching in the LASSI-L provides a comprehensive approach for evaluating various cognitive domains, including psychomotor speed, interference, perseveration, and source retrieval, which have been identified as weaknesses in preHD. This straightforward paradigm offers a practical means to evaluate these cognitive aspects simultaneously.

### Limitations

This study has potential limitations. Although the primary findings outlined in Sierra et al. (17) were replicated in a larger dataset and across different sites, examiners were not blinded to group status (preHD vs. HC). Although the PSI section appeared sensitive to early changes the LASSI-L’s overall prognostic value for future disease progression in HD remains unknown. Given that the LASSI-L was administered as part of a larger battery, assessment order or subject fatigue could have influenced the results. The typical age of onset of motor manifest HD is 30–59 (48, 49), however, the LASSI-L was initially developed for MCI/dementia and normative scores for age < 60 have not yet been established. Although neither age or education were significant confounds for PSI using ANCOVA, observed group differences could have been influenced by demographic factors not accounted for in the matching paradigm. Finally, although 50 preHD subjects were enrolled across two sites, this study does not currently meet the sample size requirement to be included as a landmark assessment in the HD-ISS staging system. The administration of the LASSI-L to even larger preHD cohorts in blinded fashion using a different order of assessment would help address many of these potential concerns.

## Conclusion

The LASSI-L is a sensitive, reliable and efficient tool for the early detection of cognitive impairment in preHD. It is simple to administer, outperforms other cognitive tests, is not associated with clear practice effects and demonstrates evidence of construct validity. With further longitudinal validation, it could be a useful addition to current research and clinical trial protocols aiming to reliably capture the earliest cognitive changes in pre-motor manifest HD.

## Data availability statement

The raw data supporting the conclusions of this article will be made available by the authors, without undue reservation.

## Ethics statement

The studies involving human participants were reviewed and approved by Beth Israel Deaconess Medical Center Institutional Review Board. The patients/participants provided their written informed consent to participate in this study.

## Author contributions

All authors listed have made a substantial, direct, and intellectual contribution to the work and approved it for publication.

## Funding

This work was supported by the Huntington’s disease Society of America HDSA Human Biology Project.

## Conflict of interest

The authors declare that the research was conducted in the absence of any commercial or financial relationships that could be construed as a potential conflict of interest.

## Publisher’s note

All claims expressed in this article are solely those of the authors and do not necessarily represent those of their affiliated organizations, or those of the publisher, the editors and the reviewers. Any product that may be evaluated in this article, or claim that may be made by its manufacturer, is not guaranteed or endorsed by the publisher.

## Footnotes

^1^http://surfer.nmr.mgh.harvard.edu
