# Supplementary figures and images for: LASSI-L detects early cognitive changes in pre-motor manifest Huntington’s disease: a replication and validation study
Source: Front Neurol. 2023 Jul 18;14:1191718. doi: 10.3389/fneur.2023.1191718 (PMC10393264; doi:10.3389/fneur.2023.1191718)

**Supplemental Figure 1: Pilot Cutoff Analysis**

**
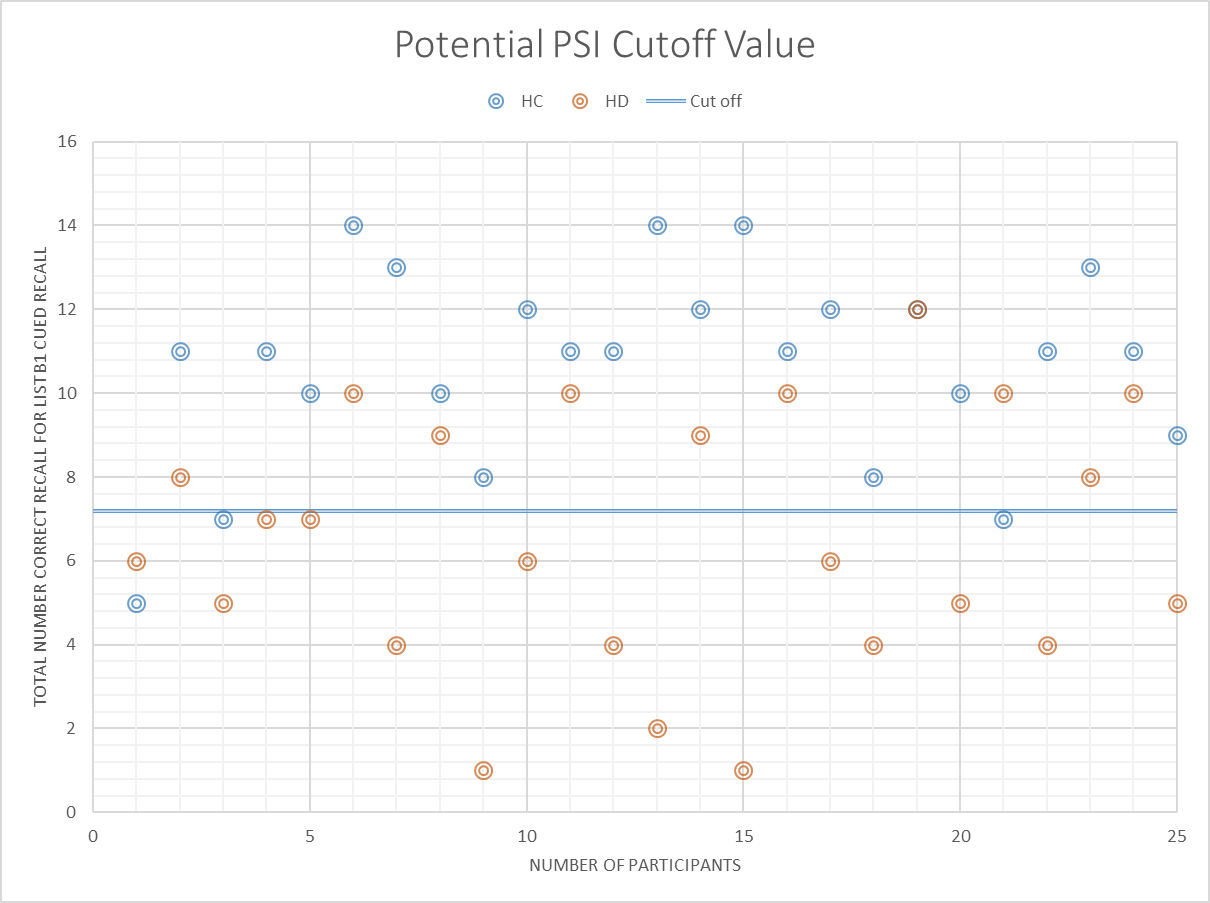
**

Supplement: Supplementary file 1 [file Data_Sheet_1.docx]
