# Supplementary material for: LASSI-L detects early cognitive changes in pre-motor manifest Huntington’s disease: a replication and validation study
Source: Front Neurol. 2023 Jul 18;14:1191718. doi: 10.3389/fneur.2023.1191718 (PMC10393264; doi:10.3389/fneur.2023.1191718)
Supplement: Supplementary file 2 [file Table_1.docx]

**Supplemental Table 1**

| **Regional brain volume correlations to selected cognitive test sections** | | | | | |  |
| --- | --- | --- | --- | --- | --- | --- |
|  | Spearman rho  (p value) | | | | | |
|  | **PSI** | **B1 Cued Recall Intrusions** | **Delayed Recall** | **PIE** | **SDMT** | **Stroop Word Reading** |
| Caudate | 0.130 (0.543) | **- 0.512 (0.010)** | 0.060 (0.780) | - 0.397 (0.055) | 0.301 (0.152) | 0.015 (0.944) |
| Putamen | - 0.082 (0.702) | - 0.340 (0.053) | - 0.87 (0.174) | - 0.397 (0.055) | 0.272 (0.199) | 0.015 (0.944) |
| Globus Pallidus | 0.010 (0.645) | - 0.271 (0.199) | - 0.120 (0.576) | -0.218 (0.306) | 0.104 (0.491) | 0.148 (0.491) |
| Accumbens | - 0.033 (0.878) | - 0.306 (0.145) | - 0.120 (0.576) | - 0.218 (0.306) | 0.357 (0.086) | 0.254 (0.232) |
